# Effects of Chicken Protein Hydrolysate Supplementation Alone or Combined With Concurrent Training on Arterial Stiffness in Adults With Hypertension: A Randomized Controlled Trial

**DOI:** 10.1155/ijhy/8962082

**Published:** 2026-08-30

**Authors:** Piyaporn Tumnark, Sarunrat Hirunrattanathum, Pakaporn Sathalalai, Tantawan Pirak, Jatuporn Phoemsapthawee

**Affiliations:** ^1^ Department of Sports Science, Faculty of Sports and Health Science, Kasetsart University, Nakhon Pathom 73140, Thailand, ku.ac.th; ^2^ Regional Sports Science Section, Sport Authority of Thailand Region 3, Nakhon Ratchasima 30000, Thailand; ^3^ Central Laboratory and Greenhouse Complex, Research and Academic Service Center, Faculty of Agriculture at Kamphaeng Saen, Kasetsart University, Nakhon Pathom 73140, Thailand, ku.ac.th; ^4^ Department of Product Development, Faculty of Agro-Industry, Kasetsart University, Bangkok 10900, Thailand, ku.ac.th

**Keywords:** arterial stiffness, brachial–ankle pulse wave velocity, chicken protein hydrolysate, concurrent training, hypertension

## Abstract

Hypertension (HT) is associated with vascular dysfunction, chronic low‐grade inflammation, and increased arterial stiffness, all of which contribute to elevated cardiovascular risk. Lifestyle‐based interventions incorporating nutritional and exercise strategies may provide complementary vascular benefits. This study investigated the effects of chicken protein hydrolysate supplementation, administered alone or combined with concurrent training (CT), on arterial stiffness, blood pressure (BP), inflammatory markers, and physical fitness in adults with HT. In this randomized controlled trial, adults with HT who completed the study were included in the analysis: control (CON, n = 12), chicken protein hydrolysate supplementation alone (PRO, n = 10; 28 g/day), and supplementation combined with CT (PRO + CT, n = 12) following the 8‐week intervention. BP, brachial–ankle pulse wave velocity (baPWV), high‐sensitivity C‐reactive protein (hs‐CRP), and physical fitness outcomes—including peak oxygen consumption (V̇O_2_peak) and upper‐ and lower‐body muscular endurance—were assessed at baseline and postintervention. Significant group × time interactions were observed for baPWV (*p* < 0.01), hs‐CRP (*p* < 0.01), V̇O_2_peak (*p* < 0.01), and upper‐ and lower‐body muscular endurance (*p* < 0.01). Both PRO and PRO + CT demonstrated greater reductions in baPWV compared with CON (*p* < 0.05). Significant reductions in hs‐CRP were observed in both PRO and PRO + CT groups (*p* < 0.01); however, only PRO + CT demonstrated a significantly greater reduction compared with CON (*p* < 0.05). Improvements in V̇O_2_peak and muscular endurance occurred exclusively in the PRO + CT group (*p* < 0.01), whereas changes in BP were modest across intervention groups. Chicken protein hydrolysate supplementation, particularly when combined with CT, was associated with improvements in arterial stiffness, while the combined intervention further improved inflammatory status and physical fitness outcomes. These findings suggest a potential role for combined nutritional and exercise‐based lifestyle interventions in improving vascular function in adults with HT.

**Trial Registration:** Thai Clinical Trials Registry: TCTR20251210008

## 1. Introduction

Hypertension (HT) is a major cardiovascular risk factor associated with progressive vascular dysfunction, chronic low‐grade inflammation, and increased arterial stiffness [1, 2]. These pathophysiological alterations contribute to endothelial impairment and accelerate cardiovascular morbidity and mortality. Among vascular indices, brachial–ankle pulse wave velocity (baPWV) is a validated and widely used marker of arterial stiffness that independently predicts cardiovascular events and mortality in individuals with HT [3–5]. Increased arterial stiffness has also been linked to inflammatory activation, including elevated high‐sensitivity C‐reactive protein (hs‐CRP), reflecting the close interaction between vascular dysfunction and systemic inflammation [6–9].

Lifestyle‐based interventions are recommended as first‐line strategies for the management of HT and vascular dysfunction [10, 11]. Concurrent training (CT), which combines aerobic and resistance exercise, has demonstrated beneficial effects on vascular function, blood pressure (BP), and cardiometabolic health [12, 13]. Aerobic exercise improves endothelial function and vascular responsiveness, whereas resistance exercise contributes to muscular fitness and metabolic regulation [12, 13]. Consistent with these physiological adaptations, previous studies, including our own, have shown that CT improves BP regulation, arterial stiffness, inflammatory status, and cardiometabolic profiles in individuals with prehypertension, HT, and other conditions associated with elevated cardiovascular risk [14–19].

Nutritional interventions may further support vascular improvements associated with exercise training. Chicken protein hydrolysate has recently emerged as a potential functional nutritional supplement due to its bioactive peptide content [20–22]. Experimental and limited clinical evidence suggests that protein hydrolysates may exert anti‐inflammatory and vascular‐protective effects, potentially through improvements in endothelial function [23–27]. However, the vascular effects of chicken protein hydrolysate in adults with HT remain poorly understood, and no previous clinical study has evaluated its effects on arterial stiffness using baPWV.

Combining nutritional supplementation with structured exercise training may provide complementary vascular benefits in adults with HT. However, evidence regarding the combined effects of chicken protein hydrolysate supplementation and CT on arterial stiffness and inflammation remains limited. Therefore, this randomized controlled trial investigated the effects of chicken protein hydrolysate supplementation, administered alone or combined with CT, on arterial stiffness and BP in adults with HT. Secondary outcomes included inflammatory markers and physical fitness outcomes. We hypothesized that both intervention groups would improve vascular outcomes compared with the control group, with potentially greater improvements following the combined intervention.

## 2. Materials and Methods

### 2.1. Study Design

This study was an 8‐week, randomized controlled clinical trial conducted at the Faculty of Sports and Health Science, Kasetsart University, Thailand, between January and June 2021. A three‐arm, parallel‐group design was employed to examine the effects of chicken protein hydrolysate supplementation administered alone or in combination with CT on arterial stiffness, BP, inflammatory markers, and physical fitness outcomes in adults with HT. The protocol complied with the Declaration of Helsinki and was approved by the Kasetsart University Research Ethics Committee (approval No. COA63/062; 23 November 2020). Although the trial was retrospectively registered, the study protocol, intervention procedures, and outcome assessments were finalized before participant enrollment and remained unchanged throughout the trial.

**FIGURE 1:**
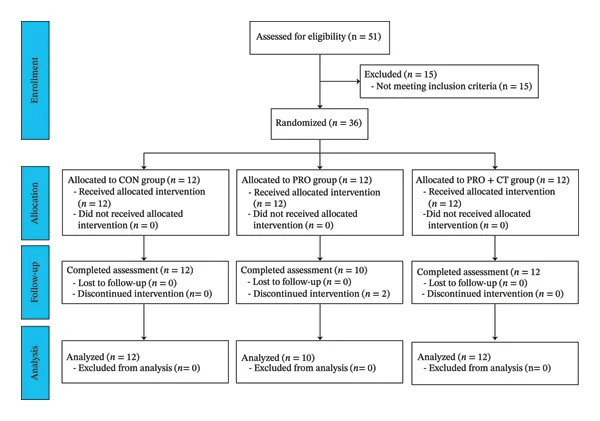
CONSORT flow diagram of the study.

Figure 1 presents the CONSORT flow diagram, with the corresponding CONSORT 2025 checklist provided in Supporting Table S1. Fifty‐one individuals were screened for eligibility, of whom 36 met the inclusion criteria and were randomized into three groups: (i) placebo control (CON; *n* = 12); (ii) chicken protein hydrolysate supplementation (PRO; *n* = 12); and (iii) combined supplementation with CT (PRO + CT; *n* = 12). Reasons for exclusion included not meeting the diagnostic criteria for HT, the presence of uncontrolled comorbidities, or declining participation. During the intervention, two participants in the PRO group did not complete the intervention and were excluded from the final analyses, resulting in final sample sizes of CON (*n* = 12), PRO (*n* = 10), and PRO + CT (*n* = 12). No adverse events, allergic reactions, or exercise‐related injuries were reported throughout the study.

Participants in the PRO and PRO + CT groups consumed crackers containing the chicken protein hydrolysate supplement, providing approximately 28 g of protein per day [28], whereas the CON group consumed isocaloric, visually indistinguishable crackers without the supplement. Only the PRO + CT group participated in structured CT, performed three times per week for 60 min per session under instructor supervision. All participants were instructed to maintain their usual physical activity levels and dietary patterns outside the intervention. Adherence and dietary intake were monitored using self‐reported nutrition logs. Adverse events, allergic reactions, and exercise‐related injuries were monitored throughout the intervention period.

Outcome assessments were conducted at baseline (within 1 week prior to intervention) and after the 8‐week intervention. To minimize the acute effects of exercise on outcome measurements, all postintervention assessments for the PRO + CT group were obtained at least 72 h after the final training session. All assessments were completed across two laboratory visits at the Faculty of Sports and Health Science, with testing standardized to the same time of day (±1 h) under controlled environmental conditions (23°C ± 2°C; 50% ± 4% humidity).

### 2.2. Participants

#### 2.2.1. Sample Size Calculation

The primary outcome of this study was baPWV. An a priori sample size calculation was performed using G^∗^ Power version 3.1.9.7 (Heinrich Heine University Düsseldorf, Germany) for a repeated‐measures analysis of variance (ANOVA) with a within–between interaction. Based on previous studies demonstrating clinically meaningful improvements in baPWV following combined exercise training [17, 29], a conservative effect size of 0.30 (Cohen’s *f*) was assumed. Using an alpha level of 0.05, a statistical power of 80%, three intervention groups, two measurement time points, a correlation among repeated measures of 0.50, and a nonsphericity correction of 1.0, the required sample size was estimated to be 30 participants. To allow for an anticipated dropout rate of 20%, a total of 36 participants were recruited.

#### 2.2.2. Inclusion and Exclusion Criteria

Eligible participants were community‐dwelling adults aged 35–59 years who met the diagnostic criteria for HT, defined as systolic blood pressure (SBP) of 130–159 mmHg and/or diastolic blood pressure (DBP) of 80–99 mmHg, confirmed on at least two separate occasions [11]. Additional eligibility criteria included a body mass index (BMI) ≥ 23 kg/m^2^ and a sedentary lifestyle, defined as engaging in < 1 h of structured exercise per week [30]. Participants were also required to have stable antihypertensive medication use, if applicable, for at least 3 months and no involvement in supervised exercise or nutrition programs during the preceding 3 months. All participants were expected to comply with study procedures, including supplementation and—when applicable—attendance at supervised CT sessions.

Individuals were excluded if they had conditions that could compromise safety or confound study outcomes, including current smoking; a history of type 2 diabetes, coronary artery disease, peripheral vascular disease, stroke, or uncontrolled HT (≥ 160/100 mmHg); musculoskeletal disorders limiting participation in moderate‐intensity exercise; psychiatric or cognitive conditions that could interfere with study participation or adherence; or clinically significant renal, hepatic, or hematologic disease. Additional exclusions included the use of glucose‐lowering agents, anti‐inflammatory medications, or dietary supplements known to influence vascular or metabolic function, as well as pregnancy or lactation.

#### 2.2.3. Randomization, Allocation, and Blinding

Following baseline evaluations, participants were randomly allocated to one of three groups: CON, PRO, or PRO + CT. Randomization was generated using computer software by an independent researcher who was not involved in recruitment, assessments, or intervention delivery. Allocation concealment was maintained using sequentially numbered, opaque, sealed envelopes prepared by an external assistant. All participants consumed crackers identical in appearance; the PRO and PRO + CT groups received crackers containing chicken protein hydrolysate, whereas the CON group received isocaloric crackers without the supplement. Participants, outcome assessors, and data analysts were blinded to supplementation allocation throughout the study. Exercise trainers were aware of group allocation only for participants assigned to the CT intervention to ensure safe and appropriate supervision of the exercise sessions.

### 2.3. Experimental Procedures

All experimental procedures were conducted under standardized laboratory conditions at the Faculty of Sports and Health Science. Participants arrived in the morning following a 12 h overnight fast and abstinence from caffeine, alcohol, and vigorous physical activity prior to testing. To minimize interobserver variability, all measurements at both time points were performed by the same trained investigators. Baseline assessments were completed across two laboratory visits within 1 week before the intervention. During Visit 1, fasting blood samples were collected, followed by anthropometric measurements, body composition analysis, resting BP measurement, and arterial stiffness assessment. Participants were subsequently familiarized with the muscle endurance and incremental exercise testing procedures. During Visit 2, standardized muscle endurance testing was performed, followed by assessment of cardiorespiratory fitness.

**FIGURE 2:**
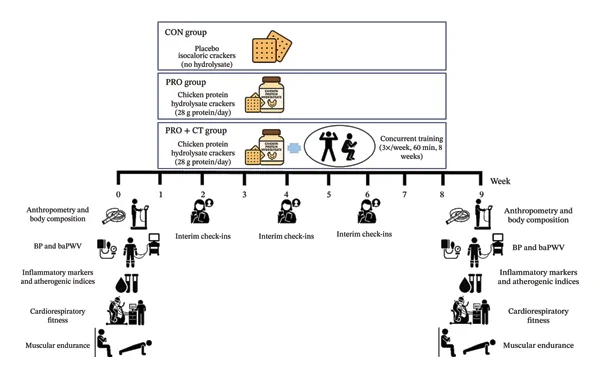
A schematic overview of the experimental procedures.

During the intervention period, participants followed their assigned supplementation and training protocols. Participants were instructed to maintain their usual dietary patterns and physical activity habits outside the study protocol. Supplement adherence and dietary intake were monitored using self‐reported dietary logs. Postintervention assessments followed the same procedures as baseline. To minimize the acute effects of exercise on outcome measurements, all assessments for the PRO + CT group were obtained at least 72 h after the final training session. Testing was conducted at the same time of day (±1 h) under identical environmental conditions to minimize potential diurnal variation. A schematic overview of the experimental timeline is presented in Figure 2.

### 2.4. Preparation of Chicken Protein Hydrolysate and Cracker Formulation

Chicken protein hydrolysate was produced at the FISP Pilot Plant, Thailand Institute of Scientific and Technological Research, Thailand, using a commercial enzymatic hydrolysis process as previously described [31]. The hydrolysate solution was collected, then freeze‐dried at −80°C under vacuum and stored at −20°C until further analysis. High‐performance liquid chromatography confirmed the presence of several amino acids, including arginine, glutamic acid, leucine, and lysine.

Chicken protein hydrolysate crackers used in the intervention were produced at the processing pilot plant, Faculty of Agriculture, Kasetsart University (Kamphaeng Saen Campus), to ensure standardized nutrient composition and consistent dosing. Each serving provided 352.7 kcal, 34.2 g carbohydrate, 28.1 g protein, and 11.5 g fat. Placebo crackers were formulated to match the carbohydrate and fat content of the intervention crackers while excluding the hydrolysate to preserve blinding across groups. Detailed nutritional and amino acid compositions of the cracker formulations are provided in Supporting Table S2.

### 2.5. Exercise Training Protocol

The CT program for the PRO + CT group was administered three times per week in supervised 60‐min sessions. Each session adhered to a standardized structure consisting of a dynamic warm‐up, a resistance training component followed by an aerobic training component, and a static stretching cool‐down. The program was adapted from established CT protocols [14, 15] and demonstrated satisfactory content validity (IOC = 0.81). Throughout all sessions, exercise intensity and participant safety were continuously monitored using a heart rate (HR) monitoring device (Polar Inc., Kempele, Finland). Each training session began with a 10‐min dynamic warm‐up consisting of light jogging, lateral leg swings, trunk rotations, arm swings, and side stretches, all of which were selected to progressively elevate HR, enhance joint mobility, and prepare the neuromuscular system for the ensuing workload.

During the first four weeks, participants completed two sets of 12 repetitions of squats, elbow lifts, deadlifts, push‐ups, and glute bridges, together with two sets of a 30‐s static plank. During weeks 5–8, exercise progression was achieved by modifying exercise variations and increasing the number of repetitions from 12 to 15 per set for the dynamic resistance exercises while maintaining two sets per exercise. All resistance exercises were performed through a full, pain‐free range of motion while maintaining proper exercise technique. Dynamic resistance exercises were performed using a controlled movement cadence of approximately 2 s for the concentric phase and 2 s for the eccentric phase [32]. A 60‐s rest interval was provided between sets and between consecutive exercises. Exercise intensity was monitored throughout the resistance training sessions by certified exercise specialists according to each participant’s ability to complete the prescribed repetitions while maintaining proper technique, and exercise progression was adjusted according to individual performance. A 30‐s transition interval was provided before participants progressed to the aerobic training component.

The aerobic component consisted of 20 min of rhythmic whole‐body movements, including marching, step touches, light walking, straight punches, uppercuts, elbow strikes, knee strikes, and high kicks. Exercise intensity was progressively increased across the 8‐week intervention, beginning at 65%–70% of heart rate reserve (HRR) during weeks 1–4 and increasing to 70%–75% HRR during weeks 5–8. HR was continuously monitored throughout the aerobic component to ensure that participants exercised within the prescribed target HRR. At the end of each session, participants completed a 10‐min cool‐down consisting of static stretching exercises held for 15–30 s and repeated twice to promote flexibility and facilitate postexercise recovery. All participants had prior experience with resistance training and received standardized instructions before each session to ensure correct exercise technique and minimize injury risk.

### 2.6. Outcome Measurements

#### 2.6.1. Anthropometry and Body Composition

Height and body weight were measured using standardized procedures, and BMI was calculated as weight (kg) divided by height squared (m^2^). Waist and hip circumferences were assessed using a nonelastic tape measure. Body composition was evaluated using multifrequency bioelectrical impedance analysis (InBody 720, Biospace Inc., Seoul, Korea). All assessments were performed in the morning following an overnight fast, with participants wearing light clothing and barefoot.

#### 2.6.2. BP

After a 5‐min seated rest period in a quiet environment, brachial BP was measured using a calibrated automatic oscillometric sphygmomanometer (Tango M2, SunTech Medical Inc., NC, USA). To minimize circadian variability, all measurements were obtained in the morning between 08:00 and 10:00. Three consecutive BP readings were recorded at 1‐min intervals, and the mean value was used for analysis [33]. SBP and DBP were recorded, and mean arterial pressure (MAP) was calculated as MAP = (SBP + 2 × DBP)/3. Additional hemodynamic indices, including the rate–pressure product (RPP = SBP × HR), were also calculated.

#### 2.6.3. Arterial Stiffness

baPWV was assessed as an index of arterial stiffness using a validated oscillometric device (BP‐203RPE III; Omron Colin Co., Tokyo, Japan) following a standardized protocol [34]. Participants rested in the supine position for at least 5 min before measurement. Four cuffs were placed bilaterally on the upper arms and ankles, and baPWV was recorded simultaneously with brachial BP, electrocardiogram, and heart sounds. The higher baPWV value from either side was used for analysis. This method has previously demonstrated high reliability and validity for the assessment of arterial stiffness [3].

#### 2.6.4. Blood Samples and Analysis

##### 2.6.4.1. Blood Sampling

After a 12 h overnight fast and at least 72 h following the final training session, venous blood samples were collected from the antecubital vein following 10 min of supine rest. Serum and plasma samples were processed and stored at −80°C until analysis. All analyses were performed in duplicate, and samples from each participant were analyzed within the same analytical batch.

##### 2.6.4.2. Analysis of Blood Chemistry and Atherogenic Indices

Fasting blood glucose (FBG) and serum lipid profiles, including total cholesterol (TC), triglycerides (TG), high‐density lipoprotein cholesterol (HDL‐c), and low‐density lipoprotein cholesterol (LDL‐c), were analyzed using automated clinical chemistry analyzers (Thermo Fisher Scientific Inc., Waltham, MA, USA). The atherogenic index of plasma (AIP), Castelli Risk Index I (CRI‐I), Castelli Risk Index II (CRI‐II), and atherogenic coefficient (AC) were subsequently calculated using standard formulas.

##### 2.6.4.3. Analysis of Inflammatory Markers

Serum hs‐CRP concentrations were measured using a particle‐enhanced immunoturbidimetric assay on an automated analyzer (Cobas c702, Roche Diagnostics, Mannheim, Germany) according to the manufacturer’s instructions. Complete blood counts were obtained using an automated hematology analyzer (DxH 800, Beckman Coulter Inc., Brea, CA, USA). The neutrophil‐to‐lymphocyte ratio (NLR), platelet‐to‐lymphocyte ratio (PLR), and systemic immune–inflammation index (SII) were calculated using standard formulas.

##### 2.6.4.4. Analysis of Electrolytes

Serum sodium (Na^+^), potassium (K^+^), and chloride (Cl^−^) concentrations were measured using an ion‐selective electrode method on an automated clinical chemistry analyzer (Cobas c702, Roche Diagnostics, Mannheim, Germany). Results are expressed in mmol/L.

#### 2.6.5. Cardiorespiratory Fitness

Participants avoided alcohol and caffeinated beverages for at least 12 h and refrained from strenuous physical activity for 48 h prior to testing. Peak oxygen consumption (V̇O_2_peak) was determined using a graded exercise test performed on an electronically braked cycle ergometer (VIAsprint 150P, Ergoline GmbH, Bitz, Germany) according to ACSM guidelines. Following a 3‐min unloaded warm‐up, exercise intensity increased incrementally beginning at 50 W and increasing by 25 W every 2 min until volitional exhaustion. Respiratory gas exchange variables were measured continuously using a calibrated metabolic system (Oxycon Mobile, CareFusion, Hochberg, Germany). V̇O_2_peak was defined as the highest oxygen uptake achieved during exercise testing.

#### 2.6.6. Muscular Endurance

Upper‐ and lower‐body muscular endurance were assessed using the push‐up test and wall sit test, respectively. For the push‐up test, men performed standard push‐ups, whereas women performed modified bent‐knee push‐ups. Participants completed as many correctly executed repetitions as possible until volitional exhaustion. Lower‐body muscular endurance was assessed using the wall sit test, during which participants maintained a 90° knee‐flexion position against a wall until exhaustion, with time recorded in seconds.

### 2.7. Statistical Analysis

All statistical analyses were performed using SPSS Version 22.0 (IBM SPSS Statistics, IBM Corp., Armonk, NY, USA). Continuous variables are presented as mean ± standard deviation (SD), while categorical variables are expressed as frequencies and percentages. Data normality was assessed using the Shapiro–Wilk test, and non‐normally distributed variables were log‐transformed prior to analysis when necessary.

Baseline differences among the three groups (CON, PRO, and PRO + CT) were evaluated using one‐way ANOVA for continuous variables and chi‐square tests for categorical variables. Intervention effects were examined using a two‐way repeated‐measures ANOVA with group (CON, PRO, and PRO + CT) and time (baseline vs. 8 weeks) as factors. Significant main effects or group × time interactions were followed by Bonferroni‐adjusted post hoc comparisons. Effect sizes were reported as partial eta squared (*η* *p* ^2^).

As a sensitivity analysis, additional analyses of covariance (ANCOVA) were performed to further evaluate the intervention effects after adjustment for potential confounding factors. Postintervention values were compared among groups using treatment group as the fixed factor, with the corresponding baseline value and baseline body weight included as covariates. Pairwise comparisons were performed using Bonferroni adjustment for multiple comparisons.

Associations among changes in BP, arterial stiffness, and inflammatory markers were examined using Pearson correlation analyses. Analyses were conducted using complete‐case data from participants who completed the study protocol and postintervention assessments. All statistical tests were two‐tailed, and statistical significance was set at *p* < 0.05.

## 3. Results

A total of 36 adults were randomized into the CON, PRO, and PRO + CT groups, of whom 34 completed the intervention and were included in the final analyses. Baseline demographic, anthropometric, and hemodynamic characteristics were comparable among groups (Table 1). Participants were predominantly female (76.5%) and middle‐aged, with a mean age of 47.6 ± 6.8 years and mean BMI of 28.5 ± 3.8 kg/m^2^, consistent with overweight or obesity according to Asian‐specific BMI criteria. Hemodynamic parameters indicated mild‐to‐moderate HT at study entry (SBP: 136.9 ± 15.4 mmHg; DBP: 84.6 ± 10.2 mmHg; MAP: 102.0 ± 11.4 mmHg). No significant between‐group differences were observed for demographic or cardiovascular variables (all *p* > 0.05), indicating comparable baseline characteristics among groups. Medication use was low and similarly distributed across groups.

**TABLE 1:** Participant baseline characteristics.

Variables	Total (*n* = 34)	CON (*n* = 12)	PRO (*n* = 10)	PRO + CT (*n* = 12)	*F*	*p* value
Age (years)	47.6 ± 6.8	49.9 ± 7.9	46.2 ± 5.3	46.4 ± 6.7	1.088	0.349
Weight (kg)	72.6 ± 9.7	74.2 ± 9.5	70.7 ± 13.0	72.7 ± 6.8	0.340	0.715
Height (cm)	159.8 ± 6.8	160.5 ± 6.7	161.0 ± 6.9	157.9 ± 7.1	0.667	0.520
BMI (kg/m^2^)	28.5 ± 3.8	28.8 ± 4.0	27.2 ± 3.5	29.3 ± 3.9	0.921	0.409
Gender (F, *n* (%))	26 (76.5%)	10 (83.3%)	7 (70.0%)	9 (75.0%)		0.755
HR (beats/min)	77.7 ± 10.3	78.4 ± 12.5	76.6 ± 8.9	77.8 ± 9.7	0.072	0.931
SBP (mmHg)	136.9 ± 15.4	134.6 ± 16.7	132.4 ± 7.3	142.8 ± 18.1	1.499	0.239
DBP (mmHg)	84.6 ± 10.2	83.2 ± 10.5	81.9 ± 6.5	88.2 ± 12.0	1.221	0.309
MAP (mmHg)	102.0 ± 11.4	100.3 ± 12.2	98.7 ± 5.8	106.4 ± 13.3	1.488	0.242
PP (mmHg)	52.3 ± 9.2	51.4 ± 9.3	50.5 ± 7.3	54.7 ± 10.8	0.625	0.542
RPP (mmHg × beats/min) × 10^3^	10.6 ± 1.7	10.5 ± 1.7	10.1 ± 1.1	11.1 ± 2.0	0.937	0.403
Medications *n* (%)	8 (23.5%)	2 (16.7%)	3 (30.0%)	3 (25.0%)		0.755
Angiotensin II receptor blockers *n* (%)	4 (11.8%)	1 (8.3%)	2 (20.0%)	1 (8.3%)		0.630
Calcium channel blockers *n* (%)	4 (11.8%)	2 (16.7%)	1 (10.0%)	1 (8.3%)		0.801
Diuretics *n* (%)	2 (5.9%)	0 (0.0%)	0 (0.0%)	2 (16.7%)		0.143
Beta‐blockers *n* (%)	1 (2.9%)	0 (0.0%)	1 (10.0%)	0 (0.0%)		0.290

*Note:* Data are presented as mean ± SD, frequency (*n*), or percentage (%).CON, placebo control group; PRO, chicken protein hydrolysate supplementation group; PRO + CT, combined chicken protein hydrolysate supplementation with concurrent training group.

Abbreviations: BMI, body mass index; DBP, diastolic blood pressure; HR, heart rate; MAP, mean arterial pressure; PP, pulse pressure; RPP, rate–pressure product; SBP, systolic blood pressure.

Dietary intake patterns remained stable throughout the intervention, with no significant differences in energy or macronutrient intake between groups or across time points, as presented in Supporting Table S3. Habitual physical activity levels outside the prescribed intervention also remained unchanged. Exercise adherence in the PRO + CT group was high, with attendance rates of 97.6% ± 5.2% for aerobic training and 96.2% ± 6.5% for resistance training. Target exercise intensity was consistently achieved throughout the intervention. No adverse events or supplement‐related side effects were reported.

### 3.1. BP and Arterial Stiffness

**FIGURE 3:**
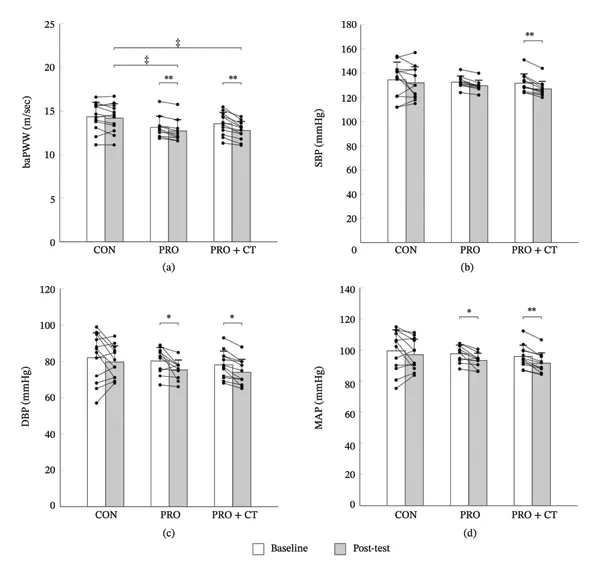
Changes in arterial stiffness and hemodynamic parameters following the 8‐week intervention: (a) baPWV; (b) SBP; (c) DBP; and (d) MAP. Data are presented as mean ± SD. Abbreviations: CON, placebo control group; PRO, chicken protein hydrolysate supplementation; PRO + CT, combined supplementation with concurrent training; baPWV, brachial–ankle pulse wave velocity; SBP, systolic blood pressure; DBP, diastolic blood pressure; MAP, mean arterial pressure. ^∗^ *p* < 0.05, ^∗∗^ *p* < 0.01 vs. baseline within group; ^‡^ *p* < 0.05 vs. CON at postintervention.

BP and arterial stiffness outcomes following the intervention are presented in Figure 3. baPWV demonstrated a significant main effect of time (*F*_(1,  31)_ = 42.76, *p* < 0.001, *η* *p* ^2^ = 0.580) as well as a significant group × time interaction (*F*_(2,  31)_ = 6.97, *p* = 0.003, *η* *p* ^2^ = 0.310). Post hoc analyses revealed significant reductions in baPWV in both the PRO and PRO + CT groups (*p* < 0.01), whereas no significant change was observed in the CON group. Reductions in baPWV were greater in the intervention groups than in CON (*p* < 0.05).

SBP and DBP also demonstrated significant time effects (SBP: *F*_(1,  31)_ = 12.79, *p* = 0.001, *η* *p* ^2^ = 0.292; DBP: *F*_(1,  31)_ = 12.42, *p* = 0.001, *η* *p* ^2^ = 0.286), although no significant group × time interactions were detected. Significant reductions in SBP were observed only in PRO + CT (*p* < 0.01), whereas DBP decreased significantly in both PRO and PRO + CT (*p* < 0.05). No significant changes were observed in the CON group. Similarly, MAP and RPP demonstrated significant time effects (MAP: *F*_(1,  31)_ = 17.13, *p* < 0.001, *η* *p* ^2^ = 0.356; RPP: *F*_(1,  31)_ = 9.45, *p* = 0.004, *η* *p* ^2^ = 0.234), without significant interaction effects. MAP decreased significantly in both PRO (*p* < 0.05) and PRO + CT (*p* < 0.01), whereas RPP decreased significantly only in PRO + CT (*p* < 0.05). No significant changes were observed in the CON group.

As a sensitivity analysis, ANCOVA was performed to examine whether adjustment for baseline body weight influenced the intervention effects. After adjustment for baseline body weight and baseline baPWV, the between‐group difference in postintervention baPWV remained statistically significant (*F*_(2,  29)_ = 8.599, *p* = 0.001, *η* *p* ^2^ = 0.372). Bonferroni‐adjusted pairwise comparisons demonstrated that the PRO + CT group had significantly lower postintervention baPWV than the CON group (mean difference = −0.679 m/s, *p* = 0.001), whereas no significant differences were observed between the PRO and CON groups or between the PRO and PRO + CT groups. In contrast, no significant between‐group differences were observed for SBP, DBP, MAP, or RPP (all *p* > 0.05).

### 3.2. Inflammatory and Immune–Inflammatory Indices

**FIGURE 4:**
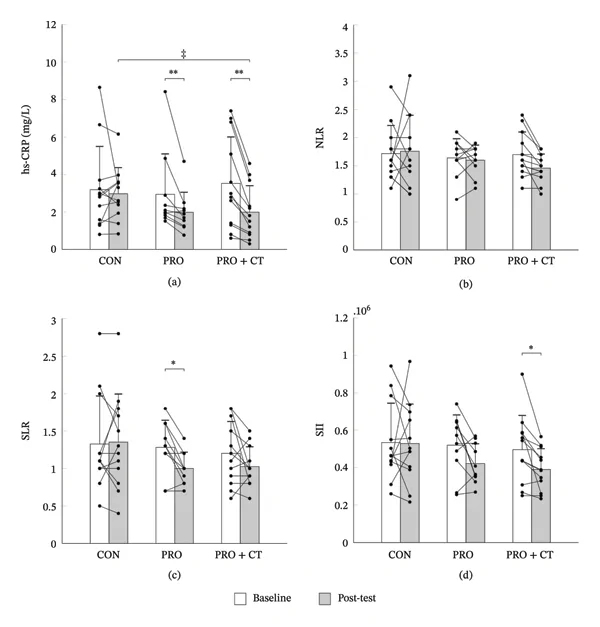
Changes in inflammatory and immune–inflammatory indices following the 8‐week intervention: (a) hs‐CRP; (b) NLR; (c) PLR; and (d) SII. Data are presented as mean ± SD. Abbreviations: CON, placebo control group; PRO, chicken protein hydrolysate supplementation; PRO + CT, combined supplementation with concurrent training; hs‐CRP, high‐sensitivity C‐reactive protein; NLR, neutrophil‐to‐lymphocyte ratio; PLR, platelet‐to‐lymphocyte ratio; SII, systemic immune–inflammation index. ^∗^ *p* < 0.05, ^∗∗^ *p* < 0.01 vs. baseline within group; ^‡^ *p* < 0.05 vs. CON at postintervention.

Inflammatory and immune–inflammatory outcomes following the intervention are presented in Figure 4. hs‐CRP demonstrated a significant main effect of time (*F*_(1,  31)_ = 25.887, *p* < 0.001, *η* *p* ^2^ = 0.455) and a significant group × time interaction (*F*_(2,  31)_ = 9.513, *p* = 0.001, *η* *p* ^2^ = 0.380). Post hoc analyses revealed significant reductions in hs‐CRP from baseline in both the PRO and PRO + CT groups (*p* < 0.001), whereas no significant change was observed in the CON group. Furthermore, only the PRO + CT group demonstrated a significantly greater reduction in hs‐CRP compared with the CON group (*p* < 0.05).

PLR demonstrated a significant main effect of time (*F*_(1,  31)_ = 5.346, *p* = 0.028, *η* *p* ^2^ = 0.147), although no significant group × time interaction was detected. A significant decrease was observed only in the PRO group (*p* < 0.05), although this reduction did not differ significantly from CON. Similarly, SII demonstrated a significant main effect of time (*F*_(1,  31)_ = 6.027, *p* = 0.020, *η* *p* ^2^ = 0.163) without a significant interaction effect. Post hoc analyses showed a significant reduction only in the PRO + CT group (*p* < 0.05), although this change did not differ significantly from CON. In contrast, NLR did not demonstrate significant main or interaction effects, indicating no significant changes across the intervention period.

A trend toward a positive association was observed between changes in hs‐CRP and SBP in the PRO + CT group (*r* = 0.537, *p* = 0.07). A significant positive association was observed between changes in baPWV and SBP in the PRO group (*r* = 0.620, *p* < 0.05).

### 3.3. Atherogenic Indices and Electrolytes

Atherogenic indices and electrolyte outcomes are presented in Table 2. CRI‐II demonstrated a significant main effect of time (*F*_(1,  31)_ = 4.450, *p* = 0.043, *η* *p* ^2^ = 0.126). Within‐group analyses indicated a reduction in CRI‐II in the PRO + CT group (*p* < 0.05), although no significant group × time interaction was observed.

**TABLE 2:** Atherogenic indices, electrolyte profiles, and physical fitness parameters at baseline and after 8 weeks of intervention.

	CON (*n* = 12)		PRO (*n* = 10)		PRO + CT (*n* = 12)		Time effect *η* *p* ^2^ (*p* value)	Group × time interaction *η* *p* ^2^ (*p* value)
	Baseline	Post‐test	Baseline	Post‐test	Baseline	Post‐test		
Atherogenic indices								
AIP	0.6 ± 0.2	0.6 ± 0.1	0.5 ± 0.3	0.5 ± 0.2	0.6 ± 0.3	0.6 ± 0.2	0.018 (0.458)	0.115 (0.149)
CRI‐I	5.7 ± 1.2	5.6 ± 1.0	5.4 ± 1.0	5.4 ± 1.2	5.4 ± 1.2	4.8 ± 1.3	0.076 (0.121)	0.101 (0.193)
CRI‐II	3.7 ± 1.0	3.4 ± 0.8	3.6 ± 0.8	3.5 ± 0.9	3.3 ± 1.2	2.9 ± 1.2^a∗^	0.126 (0.043)^†^	0.055 (0.416)
AC	4.7 ± 1.2	4.6 ± 1.0	4.4 ± 1.0	4.4 ± 1.2	4.3 ± 1.3	3.8 ± 1.3	0.081 (0.108)	0.098 (0.203)
Electrolyte profiles								
Sodium (mmol/L)	138.1 ± 3.3	139.0 ± 2.8	136.1 ± 3.2	138.2 ± 3.2	138.2 ± 4.3	138.4 ± 2.2	0.081 (0.108)	0.040 (0.531)
Potassium (mmol/L)	4.3 ± 0.3	4.4 ± 0.1	4.3 ± 0.3	4.2 ± 0.5	4.2 ± 0.3	4.4 ± 0.1^a∗∗^	0.299 (0.001)^††^	0.025 (0.680)
Chloride (mmol/L)	103.0 ± 2.7	100.8 ± 1.8	104.7 ± 1.5	100.5 ± 2.1^a∗∗^	104.0 ± 1.8	102.1 ± 1.9^a∗∗^	0.630 (0.001)^††^	0.137 (0.102)
Physical fitness								
V̇O_2_peak (mL/kg/min)	28.0 ± 6.0	27.7 ± 5.6	27.8 ± 5.5	28.1 ± 5.6	26.6 ± 3.8	30.6 ± 3.5^a∗∗,b∗∗^	0.219 (0.006)^††^	0.359 (0.001)^††^
Push‐up test (reps)	3.1 ± 3.2	4.1 ± 4.0	7.8 ± 9.0	8.5 ± 8.5	6.3 ± 6.3	17.1 ± 8.9^a∗∗,b∗∗^	0.440 (0.001)^††^	0.507 (0.001)^††^
Wall sit test (s)	46.0 ± 30.9	35.9 ± 12.2	42.7 ± 20.2	37.2 ± 17.3	38.4 ± 11.4	57.4 ± 23.8^a∗∗,b∗^	0.185 (0.013)^††^	0.274 (0.007)^††^

*Note:* Data are expressed as mean ± SD. ^∗^ and ^∗∗^ indicate statistical significance at *p* < 0.05 and *p* < 0.01, respectively. Superscript a indicates a significant within‐group difference from baseline. Superscript b indicates a significant difference from CON. CON, placebo control group; PRO, chicken protein hydrolysate supplementation; PRO + CT, combined supplementation with concurrent training; V̇O_2_peak, peak oxygen consumption.

Abbreviations: AC, atherogenic coefficient; AIP, atherogenic index of plasma; CRI‐I, Castelli Risk Index I; CRI‐II, Castelli Risk Index II.

^†^A small‐to‐moderate effect size (0.06 ≤ partial *η* ^2^ < 0.14).

^††^A large effect size (partial *η* ^2^ ≥ 0.14).

Electrolyte variables demonstrated significant time effects for K^+^ (*F*_(1,  31)_ = 13.230, *p* = 0.001, *η* *p* ^2^ = 0.299) and Cl^−^ (*F*_(1,  31)_ = 52.867, *p* < 0.001, *η* *p* ^2^ = 0.630), without significant group × time interactions. K^+^ increased significantly only in PRO+CT (*p* < 0.01), whereas Cl^−^ decreased significantly in both PRO and PRO + CT (*p* < 0.01). No significant changes were observed for Na^+^ across groups.

No significant main effects of time or group, nor significant group × time interactions, were observed for FBG, TC, TG, HDL‐c, or LDL‐c. Detailed lipid profile outcomes from this cohort have been previously reported by our research group [35] and are therefore not presented further in the current manuscript.

### 3.4. Physical Fitness

Physical fitness outcomes are presented in Table 2. V̇O_2_peak demonstrated a significant main effect of time (*F*_(1,  31)_ = 8.695, *p* = 0.006, *η* *p* ^2^ = 0.219) and a significant group × time interaction (*F*_(2,  31)_ = 8.690, *p* = 0.001, *η* *p* ^2^ = 0.359). Post hoc analyses showed that V̇O_2_peak increased significantly only in the PRO + CT group (*p* < 0.01), which also demonstrated higher postintervention values compared with CON (*p* < 0.01).

Upper‐body muscular endurance, assessed using the push‐up test, demonstrated a significant time effect (*F*_(1,  31)_ = 24.356, *p* < 0.001, *η* *p* ^2^ = 0.440) and a significant interaction effect (*F*_(2,  31)_ = 15.965, *p* < 0.001, *η* *p* ^2^ = 0.507). Significant improvements were observed only in the PRO + CT group (*p* < 0.01), with greater improvements than CON (*p* < 0.01). Similarly, lower‐body muscular endurance, assessed using the wall‐sit test, demonstrated significant time and interaction effects (time: *F*_(1,  31)_ = 7.029, *p* = 0.013, *η* *p* ^2^ = 0.185; interaction: *F*_(2,  31)_ = 5.860, *p* = 0.007, *η* *p* ^2^ = 0.274). Significant improvements were again observed only in PRO + CT (*p* < 0.01), with greater improvements compared with CON (*p* < 0.05).

No significant changes were observed in anthropometric or body composition variables over the intervention period, with neither significant time effects nor group × time interactions detected. Detailed anthropometric and body composition outcomes from this cohort have been previously reported by our research group [35] and are therefore not presented further in the current manuscript.

## 4. Discussion

This randomized controlled trial evaluated the effects of chicken protein hydrolysate supplementation, administered alone or in combination with CT, on arterial stiffness and BP in adults with HT. The intervention yielded potentially clinically relevant reductions in baPWV, with decreases of 5.6% (−0.7 m/s) in the PRO + CT group and 3.2% (−0.4 m/s) in the PRO group, accompanied by modest reductions in SBP. These changes are clinically relevant, as a meta‐analysis has shown that each 1 m/s increase in baPWV is associated with an approximately 12% higher risk of cardiovascular disease [5], highlighting the potential clinical relevance of the baPWV reductions observed in the present study. Although SBP declined by 3–5 mmHg—an extent associated with meaningful cardiovascular risk reduction [10]—the absence of a significant group × time interaction indicates that BP changes should be interpreted cautiously. In contrast, reductions in baPWV represented the most consistent vascular adaptation associated with the interventions. Improvements in arterial stiffness also occurred in parallel with significant reductions in hs‐CRP, suggesting that attenuation of low‐grade vascular inflammation may have contributed to enhanced arterial compliance.

These inflammatory improvements are consistent with physiological adaptations associated with CT, which increases endothelial shear stress and has been linked to improved nitric oxide (NO) bioavailability, reduced oxidative stress, and improved autonomic function—mechanisms that may contribute to reductions in arterial stiffness and BP in individuals with prehypertension and HT [14–17, 36]. HT is associated with early vascular dysfunction characterized by increased inflammatory activity and arterial stiffening, and hs‐CRP and pulse wave velocity are widely used indicators reflecting these alterations [6–9]. Consistent with this interpretation, evidence from meta‐analyses and meta‐regression supports the role of combined aerobic and resistance training in reducing systemic inflammation and arterial stiffness [37].

Within this context, broader immune–inflammatory indices may provide additional insight into inflammatory adaptations associated with supplementation and CT. Although changes in PLR and SII did not differ significantly between groups, their directional patterns were generally consistent with the overall anti‐inflammatory profile observed following the interventions. Specifically, PLR decreased in the PRO group, whereas SII declined in the PRO + CT group; however, these findings should be interpreted cautiously given the absence of significant group × time interactions. Such patterns are consistent with evidence indicating that repeated exercise exposure may attenuate pro‐inflammatory signaling pathways and modulate immune responses [24, 25, 38]. In contrast, the absence of change in NLR is consistent with short‐duration interventions, in which more sensitive markers such as hs‐CRP, PLR, and SII may better reflect early inflammatory modulation.

Several of the observed vascular improvements may be partly attributable to the bioactive amino acid and peptide composition of the chicken protein hydrolysate. Poultry‐derived protein hydrolysates contain arginine, which is linked to endothelial NO‐mediated vasodilation, together with branched‐chain and hydrophobic amino acid residues that may support antioxidant actions and angiotensin‐converting enzyme (ACE)–inhibitory peptide activity, thereby contributing to vascular regulation and BP control [22, 24, 25, 27]. However, these interpretations should be approached cautiously, as the present study did not directly assess peptide bioavailability, ACE activity, or molecular signaling pathways.

Evidence from preclinical studies using spontaneous hypertensive rat models provides additional biological support for the vascular responses associated with chicken protein hydrolysate. Bioactive peptides derived from chicken protein hydrolysate have been shown to exert ACE‐inhibitory and antioxidant effects and to improve vascular compliance [23, 25, 26]. Although these findings cannot be directly extrapolated to humans, they provide supportive physiological context for the vascular responses observed in the present study.

Electrolyte responses may provide additional physiological context for the vascular and hemodynamic changes observed following the intervention. The increase in serum K^+^ observed in the PRO+CT group may be relevant to vascular regulation, given the role of potassium in vascular smooth muscle membrane potential and vascular tone, and the association between higher serum K^+^ levels and favorable BP regulation [39, 40]. Reductions in serum Cl^−^ observed in both intervention groups may also reflect alterations in electrolyte and volume‐regulatory processes associated with BP regulation [41]. However, these interpretations remain speculative and should be interpreted cautiously, as renal electrolyte‐regulating pathways and vascular ion‐channel activity were not directly assessed in the present study.

In line with these findings, the largely unchanged lipid profile—apart from a modest improvement in CRI‐II in the PRO + CT group—suggests that the cardiometabolic effects of the intervention may have been mediated predominantly through vascular and inflammatory pathways rather than lipid modification.

Improvements in aerobic capacity and muscular endurance were most likely associated with the CT intervention, although the absence of a CT‐only group precludes definitive separation of the independent effects of exercise and supplementation. The increase in V̇O_2_peak in the PRO + CT group is consistent with physiological adaptations commonly associated with CT, including enhanced cardiorespiratory function, skeletal muscle oxidative capacity, and mitochondrial adaptations [42–45]. These functional improvements may also be physiologically consistent with concurrent reductions in arterial stiffness and inflammatory status, which can influence vascular function and oxygen transport capacity [46]. Similarly, upper‐ and lower‐body muscular endurance improved only in the PRO + CT group, a finding that is consistent with neuromuscular and metabolic adaptations induced by combined aerobic and resistance training [42–45]. Taken together, these findings suggest that CT likely contributed importantly to the observed improvements in physical fitness, while chicken protein hydrolysate supplementation may have provided additional supportive effects within the combined lifestyle intervention.

This study has several limitations that should be acknowledged. First, the 8‐week intervention period may have been insufficient to induce more pronounced changes in lipid profiles, body composition, or selected inflammatory markers. Second, although supplementation was blinded, the exercise intervention could not be blinded because of the nature of the intervention, which may have influenced effort‐dependent outcomes. Third, the study did not include a CT‐only group. Therefore, the independent effects of CT could not be fully distinguished from the additional effects of chicken protein hydrolysate supplementation. Future randomized controlled trials incorporating a CT‐only group are warranted to clarify the individual and combined contributions of CT and chicken protein hydrolysate supplementation to vascular health in adults with HT. Finally, these findings are generalizable only to adults with mild‐to‐moderate HT, and surrogate vascular indices such as baPWV, although clinically informative, cannot substitute for long‐term cardiovascular outcome data.

## 5. Conclusion

Chicken protein hydrolysate supplementation, particularly when combined with CT, was associated with improvements in arterial stiffness and markers of systemic inflammation in adults with HT. Reductions in baPWV emerged as the most consistent vascular response, while improvements in aerobic capacity and muscular endurance were observed exclusively with the combined intervention, likely reflecting the contribution of CT to functional adaptations. Collectively, these findings suggest that chicken protein hydrolysate may support vascular function, with CT augmenting its beneficial effects as part of a nonpharmacologic lifestyle intervention. Further studies of longer duration and with comprehensive mechanistic assessments are warranted to evaluate the long‐term persistence of these effects and clarify their relevance to cardiovascular outcomes.

## Funding

This research was funded by the Targeted Research Project Grant, Kamphaeng Saen Campus, Fiscal Year 2019.

## Disclosure

The authors reviewed and approved all revisions and accept full responsibility for the final content.

## Conflicts of Interest

The authors declare no conflicts of interest.

## Supporting Information

Additional supporting information can be found online in the Supporting Information section.

## Supporting information


**Supporting Information** The supporting information associated with this article is available online and includes Supporting Table S1. CONSORT 2025 checklist for the study. Supporting Table S2. Nutritional and amino acid compositions of the chicken protein hydrolysate cracker. Supporting Table S3. Dietary energy and macronutrient intake of participants in each group before and after the intervention.

## Data Availability

The data that support the findings of this study are available from the corresponding author upon reasonable request.
